# Survivin gene silencing sensitizes prostate cancer cells to selenium growth inhibition

**DOI:** 10.1186/1471-2407-10-418

**Published:** 2010-08-10

**Authors:** Xichun Liu, Ruijuan Gao, Yan Dong, Lifang Gao, Yanying Zhao, Lijuan Zhao, Xuejian Zhao, Haitao Zhang

**Affiliations:** 1 Prostate Diseases Prevention and Treatment Research Center and Department of Pathophysiology, Norman Bethune College of Medicine, Jilin University, Xinmin Street, Changchun, 130021, PR China; 2 Department of Pathology and Laboratory Medicine, Tulane University School of Medicine, 1430 Tulane Avenue, New Orleans, LA 70112, USA; 3 Department of Structural and Cellular Biology, Tulane University School of Medicine, 1430 Tulane Avenue, New Orleans, LA 70112, USA; 4 Molecular Signaling Program, Louisiana Cancer Research Consortium, 1615 Poydras Street, Suite 1000, New Orleans, LA 70112, USA

## Abstract

**Background:**

Prostate cancer is a leading cause of cancer-related death in men worldwide. Survivin is a member of the inhibitor of apoptosis (IAP) protein family that is expressed in the majority of human tumors including prostate cancer, but is barely detectable in terminally differentiated normal cells. Downregulation of survivin could sensitize prostate cancer cells to chemotherapeutic agents *in vitro *and *in vivo*. Selenium is an essential trace element. Several studies have shown that selenium compounds inhibit the growth of prostate cancer cells. The objective of this study is to investigate whether survivin gene silencing in conjunction with selenium treatment could enhance the therapeutic efficacy for prostate cancer and to elucidate the underlying mechanisms.

**Methods:**

Expression of survivin was analyzed in a collection of normal and malignant prostatic tissues by immunohistochemical staining. *In vitro *studies were conducted in PC-3M, C4-2B, and 22Rv1 prostate cancer cells. The effect of selenium on survivin expression was analyzed by Western blotting and semi-quantitative RT-PCR. Survivin gene knockdown was carried out by transfecting cells with a short hairpin RNA (shRNA) designed against survivin. Cell proliferation was quantitated by the 3-(4,5-Dimethylthiazol-2-yl)- 2,5-Diphenyltetrazolium Bromide (MTT) assay and apoptosis by propidium iodide staining followed by flow cytometry analysis. Finally, *in vivo *tumor growth assay was performed by establishing PC-3M xenograft in nude mice and monitoring tumor growth following transfection and treatment.

**Results:**

We found that survivin was undetectable in normal prostatic tissues but was highly expressed in prostate cancers. Survivin knockdown or selenium treatment inhibited the growth of prostate cancer cells, but the selenium effect was modest. In contrast to what have been observed in other cell lines, selenium treatment had little or no effect on survivin expression in several androgen-independent prostate cancer cell lines. Survivin knockdown sensitized these cells to selenium growth inhibition and apoptosis induction. In nude mice bearing PC-3M xenografts, survivin knockdown synergizes with selenium in inhibiting tumor growth.

**Conclusions:**

Selenium could inhibit the growth of hormone-refractory prostate cancer cells both *in vitro *and *in vivo*, but the effects were modest. The growth inhibition was not mediated by downregulating survivin expression. Survivin silencing greatly enhanced the growth inhibitory effects of selenium.

## Background

Prostate cancer (PCa) is the second most commonly diagnosed cancer and the second leading cause of cancer deaths in men in the United States [1]. Surgery and radiation therapy are effective for localized disease [2], but there is no effective treatment strategy for recurrent or metastatic PCa that has failed surgery, radiation or hormonal therapy. Chemotherapeutic drugs could only extend the lives of men with advanced prostate cancer by months, and they are also associated with dose-limiting toxicity. With the recent advances in the understanding of molecular pathways involved in prostate cancer progression, targeted therapies that are designed to interfere with the way cancer cells grow and survive offer new hope in prostate cancer therapeutics.

Survivin, a structurally unique member of the inhibitor of apoptosis (IAP) protein family, is involved in the control of mitotic progression and inhibition of apoptosis [3]. It is abundantly expressed in cancer tissues but is undetectable in normal, differentiated adult tissues. Survivin expression is associated with poor prognosis in many cancer types [4]. The expression of survivin increases gradually from normal prostate tissue, to primary low-grade prostate carcinoma, and to primary high-grade carcinoma, with the highest expression observed in foci of prostate cancer metastasized to the lymph nodes [5]. This pattern of gene expression suggests that survivin is associated with disease progression, and therefore making survivin an attractive target for prostate cancer therapeutics.

Selenium is an essential trace element. Several case control studies have demonstrated an inverse correlation between serum selenium level and the risk of developing prostate cancer [6-8]. The Nutritional Prevention of Cancer (NPC) trial demonstrated that supplementation of selenium, in the form of selenized yeast, could reduce the incidence of prostate cancer by ~50%  [9,10]. Although the interim analysis of the Selenium and Vitamin E Chemoprevention Trial (SELECT) indicated no reduction in prostate cancer risk associated with selenium supplementation [11], the finding should not be simply interpreted as selenium is ineffective against prostate cancer. In the Discussion, we provide several potential explanations for the discrepancy of the findings in SELECT and the NPC trial. The negative SELECT finding makes it more important and imperative to study the efficacy of new selenium compounds [12-14], including the compounds used in the study, methylseleninic acid (MSA) and methylselenocysteine (MSC), for prostate cancer intervention.

One of mechanisms proposed for the anticancer activity of selenium is apoptosis induction. Due to the role of survivin in conferring cytoprotection against apoptosis in cancer cells, we set out to examine the effect of selenium on the expression of survivin in several hormone refractory prostate cancer cell lines. To our surprise, selenium treatment did not affect survivin expression in these cells. Based on this finding, we hypothesize that selenium treatment and survivin knockdown would complement the action of each other, leading to a greater anticancer effect. The present study was designed to test this hypothesis, and to explore the underlying mechanisms.

## Methods

### Reagents

MSA and MSC were obtained from PharmaSe (Lubbock, TX). The rabbit anti-human polyclonal antibodies against survivin and caspase 3 were from Santa Cruz Biotech (Santa Cruz, CA). The rabbit anti-human Ki-67 polyclonal antibody was obtained from Neomarker (Fremont, CA). Trizol, RPMI 1640, fetal bovine serum (FBS), and lipofectamine 2000 were purchased from Invitrogen (Carlsbad, CA). Acridine orange (AO) and ethidium bromide (EB) were from Sigma (St. Louis, MO).

### Prostate tissue acquisition and immunohistochemical (IHC) analysis

Prostate cancer specimens were obtained from patients undergoing prostatectomy in the Second and the Third Affiliated Hospitals of Jilin University. Normal prostate tissues were obtained from patients undergoing surgery for benign prostatic hyperplasia (BPH) in these hospitals. This study was approved by the Research Ethics Committee of Norman Bethune College of Medicine, Jilin University and was in compliance with the Helsinki Declaration. The tissues were examined by the pathologists to confirm the diagnosis before IHC analyses. All specimens were fixed in 10% formalin, embedded in paraffin, and cut into 4 μm-thick slides. The slides were dewaxed, and the endogenous peroxidase activity was blocked by treatment with 3% hydrogen peroxide solution in methanol for 20 min. Epitope retrieval was performed by treating the slides with 10 mM sodium citrate buffer (pH 6.0) and heating in a microwave oven for two times at the high power for 6 min each. Non-specific binding was prevented by blocking with normal goat serum (1:10) for 10 min. Immunostaining of survivin was performed using a rabbit anti-human survivin polyclonal antibody. Goat anti-rabbit IgG conjugated with horseradish peroxidase was used as the secondary antibody. The staining procedure was carried out manually at room temperature, using an avidin-biotin-peroxidase complex method. The presence of survivin and Ki-67 was evaluated by staining with rabbit anti-survivin and anti-Ki-67 antibody, respectively. After incubation with the primary antibody for 60 min, the slides were incubated with the biotinylated goat anti-rabbit IgG (H+L) (DAKO, Carpinteria, CA) at 37°C for 30 min, followed by incubation with a 1:200 streptavidin-biotin-peroxidase complex (Sigma, St. Louis) for 30 min. Reactive products were visualized with 3,3'-diaminobenzidene (DAB) as the chromogen, and the slides were counterstained with hematoxylin and coverslipped. Sections previously known to express survivin and Ki-67 were included in each run, receiving either the primary antibody as the positive control, or a mouse IgG as the negative control. The stained slides were analyzed with a microscope at 200× magnification. The slides were examined by two independent pathologists who had no knowledge of the clinical outcomes and of the results obtained by the other. Differences in interpretation were reconciled by reviewing the slides jointly. Cellular brownish staining was scored as positive and the threshold was set at 10%. The proliferation index was defined as percent of tumor cells stained positively for Ki-67.

### Construction of the short hairpin RNA (shRNA) vectors for survivin silencing

Oligonucleotides for anti-survivin shRNA expression were designed, synthesized, and cloned into the pGCsilencer-U6/Neo/GFP plasmid (GeneChem Co, Shanghai, China). The sequences are: sense, 5'-gatcccGCAGTTTGAAGAATTAACCttcaagaga GGTTAATTCTTCAAACTGCtttttggat-3'; and antisense, 5'-agctatccaaaaaGCAGTTTGAAGAATTAACCtctcttgaaGGTTAATTCTTCAAATGCgg-3'. Sequences in uppercase letters indicate nucleotides 394-412 of the survivin cDNA. BLAST searches of the human genome database were carried out to ensure that the sequences would not target any other transcripts. The recombinant plasmid was named pGCsh-survivin (referred to as sh-survivin hereafter). Recombinant pGCsh-scrambled plasmid (referred to as sh-scrambled hereafter), which used a scrambled sequence that has no significant homology to any mouse or human gene sequences, was constructed as the negative control.

### Cell culture and transfection

The PC-3M, C4-2B, and 22Rv1 cells were cultured in RPMI 1640 medium supplemented with 10% FBS, 100 unit/ml of penicillin, 100 μg/ml of streptomycin, and 2 mM of L-glutamine and incubated at 37°C in 5% CO_2_. For transfection, 5 × 10^5 ^cells were plated onto 6-well plates 24 hours before transfection. Transfection was performed using Lipofectamine 2000 according to the manufacturer's instructions. Transfection media were removed 6 hours after transfection and replaced with fresh complete medium containing 10% FBS. Controls included lipofectamine 2000 treated cells and sh-scrambled transfected cells.

### Gene expression analysis by semi-quantitative reverse-transcription PCR (RT-PCR) and Western analysis

The effects of survivin shRNA and MSA, alone or in combination, on cellular expression of survivin were measured by semi-quantitative RT-PCR and Western blot analysis. PC-3M cells were divided into 5 groups: (a) control (mock-transfected); (b) scrambled (transfected with sh-scrambled); (c) sh-survivin (transfected with sh-survivin); (d) MSA (treated with 5 μM MSA); (e) combination (transfected with sh-survivin and treated with 5 μM MSA), and cells were harvested after 66 hours following transfection and treatment. Total RNA was isolated using the Trizol reagent according to the manufacturer's instructions. Survivin gene expression was determined by semi-quantitative RT-PCR, using the primer pairs 5'-GAATTCATGGGTGCCCCGACGTTGCC-3' and 5'-AGATCTTTCTTCTTATTGTTGGTTTCC-3'. The housekeeping gene β-actin was amplified as an internal standard.

For Western blot analysis, cells were lysed with HEPES lysis buffer (30 mM HEPES, 1% Triton X-100, 10% glycerol, 5 mM MgCl_2_, 25 mM NaF, 1 mM EDTA and 10 mM NaCl). Fifty micrograms of protein were electrophoresed on a 10% SDS-PAGE gel, transferred onto a PVDF membrane (Millipore, Bedford, MA), and probed with the anti-survivin or the anti-caspase-3 antibody. A goat anti-rabbit antibody labeled with horseradish peroxidase (Amersham) was added as the secondary antibody. Immunoblots were developed using a chemiluminescence detection system (Amersham Pharmacia Biotech).

### Cell proliferation and apoptosis assays

Cell numbers were determined at 66 hours after treatment using the 3-(4,5-dimethylthiazol-2-yl)-2, 5-diphenyltetrazolium bromide (MTT) assay and quantified by using a microplate reader (Bio-Rad). For apoptosis, 10^6 ^cells were fixed in 70% (v/v) ethanol, stained with PBS containing 50 μg/mL propidium iodide, 10 mg/mL RNase, and 0.1% (v/v) Tween-20 for 30 minutes at room temperature, and analyzed for cell apoptosis with a FACScan flow cytometer (Becton Dickinson). For AP/EB staining, PC-3M cells were collected by trypsinization, washed with PBS (pH 7.4), and fixed in 4% paraformaldehyde. Fixed cells were washed twice with PBS and resuspended in PBS containing 16 μg/ml AO and 16 μg/ml EB, then mounted on a glass slide and observed under a fluorescent microscope.

### Antitumor effect of survivin siRNA and selenium *in vivo*

Five-week-old pathogen-free athymic nude mice were purchased from the Institute of Laboratory Animal Science, Chinese Academy of Medical Sciences (Beijing, China). PC-3M cells in exponential growth phase were harvested and single-cell suspensions (2 × 10^6 ^cells in 100 μl PBS) were injected subcutaneously (s.c.) into the right dorsal flank of nude mice. Tumor size was measured every 2 to 3 days, and tumor volume calculated as 0.5236 × *width^2 ^*× *length*. After palpable tumors had developed, the mice were divided randomly into five groups (six mice per group): (a) control (mock-transfected); (b) scrambled (transfected with sh-scrambled); (c) sh-survivin (transfected with sh-survivin); (d) MSC (given MSC at 100 μg/mouse/day); (e) combination (transfected with sh-survivin and given MSC). Transfection of the siRNAs was performed by an electroporation method. Twenty micrograms of plasmid suspended in 50 μl PBS was injected percutaneously into the tumors by using a syringe with a 27-gauge needle. Immediately after injection, tumors were pulsed with an electroporation generator (ECM830, BTX, Holliston, MA). Pulses were delivered at a frequency of 1 pulse/sec, 150 V/cm, for 50 milliseconds. This process was performed twice, first on day 15 and then on day 25. For selenium treatment, MSC was given by daily intragastric administration starting at day 15. Mice were sacrificed on day 35 when tumor burden in the control group approaching 10% of body weight. Tumors were excised, fixed in 4% buffered formalin, and embedded in paraffin. The animal study was conducted following internationally recognized guidelines and was approved by the Animal Research Committee of Norman Bethune College of Medicine, Jilin University.

### Terminal deoxynucleotidyl transferase-mediated dUTP-biotin nick end labeling (TUNEL) assay

Apoptotic cells in tissues were determined by using the TUNEL assay (Roche Diagnostics, Indianapolis, IN) according to the manufacturer's instructions. Briefly, after deparaffinization and rehydration, sections were incubated with proteinase K (16.2 μg/mL in 10 mM Tris·HCL, pH 7.4) for 20 min at 37°C. Slides were rinsed with PBS and incubated with 3% H_2_O_2 _in methanol for 10 min at room temperature to block endogenous peroxidase activity, followed by PBS washing and incubation in 0.1% Triton X-100 in 0.1% sodium citrate for 2 min on ice. Sections were then incubated with a mixture of terminal deoxynucleotidyl transferase (TdT) solution and biotin-dUTP solution in a humidified chamber at 37°C for 60 min, followed by washing with PBS and incubation with peroxidase-conjugated streptavidin in a humidified chamber for 30 min at room temperature. After an additional wash with PBS, the slides were incubated in DAB and counterstained with hematoxylin. Paraffin-embedded sections of normal tonsils were used as positive control. Negative control was obtained by replacing the TdT solution with distilled water. The presence of clear nuclear staining was indicative of apoptotic cells. At least 1,000 tumor cell nuclei were randomly selected and examined. The number of TUNEL-positive tumor cell nuclei was counted and the apoptotic index was calculated as follows: apoptotic index = (number of apoptotic cells/total cell number) × 100%.

### Statistical analysis

Chi-square test was used to analyze the difference in survivin expression between normal and cancerous prostate tissues. Pearson correlation analysis was performed to test the correlation of Gleason Score and survivin expression in the prostate cancer cases. Data were presented as mean ± standard deviation (SD) when appropriate. Two-tailed *Student's t*-test was used to evaluate the differences between treatment and control values, and *P *< 0.05 was considered statistically significant.

## Results

### Survivin is overexpressed in prostate cancer tissues

**Figure 1:**
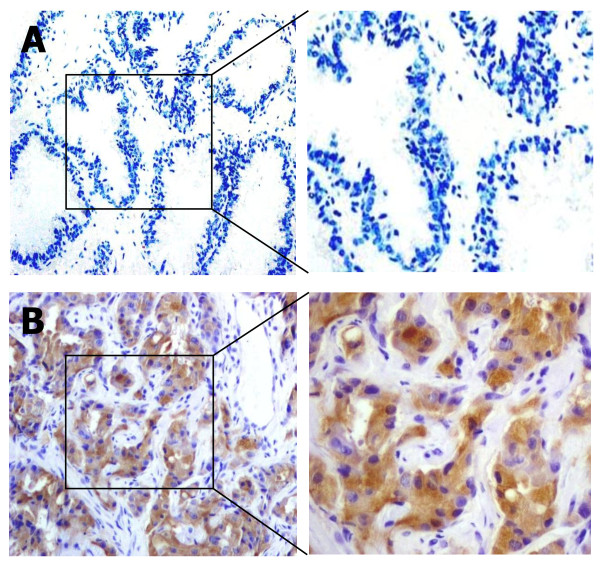
**Representative images from immunohistochemical analysis of survivin expression in normal and cancerous prostatic tissue**. A, normal prostate. B, prostate cancer. The images were obtained at 200× magnification.

We first analyzed the expression of survivin by IHC staining in a collection of prostate cancer and normal tissues in a Chinese cohort. As shown in Fig. 1, the survivin protein was virtually undetectable in normal prostatic tissue but highly expressed in prostate cancer. The cellular localization of survivin is mostly cytoplasmic, but some weak staining can be found in the nucleus. Overall, survivin was expressed in 24 of 28 (85.7%) prostate cancer specimens, and no survivin expression could be detected in 22 normal prostate tissues (Table 1). The difference is statistically significant (*P *< 0.001). Pearson correlation analysis identified a strong correlation between Gleason score and survivin expression among the cases (R = 0.7446, *P *< 0.0001). Please see additional file-1 for the original data used to perform this analysis. These results confirmed previous findings that survivin is expressed in clinical prostate cancers [5], suggesting survivin is a promising target for prostate cancer therapy.

**Table 1:** Expression of survivin in normal and cancerous prostatic tissues.

Tissue Type	survivin staining status		*P*
		
	positive N (%)	negative N (%)	
Normal	0 (0%)	22 (100%)	
			<0.001
Prostate cancer	24 (85.71%)	4 (15.29%)	

### MSA induces apoptosis in PC-3M cells but has little effect on survivin expression

**Figure 2:**
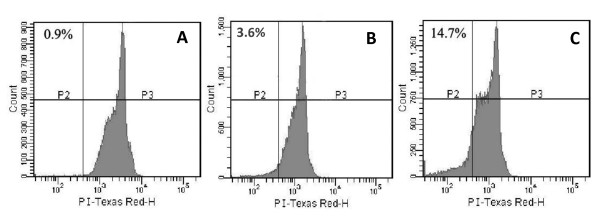
**MSA induces apoptosis in PC-3M cells**. Untreated cells (A) or cells treated with 5 μM MSA for 48 hr (B) or 72 hr (C) were labeled with propidium iodide (PI) and the histograms of DNA content were obtained by flow cytometry analysis. The x-axis indicates PI fluorescence intensity, and y-axis indicates cell number. The numbers above the histogram are percentage of cells in the sub-G1 (apoptotic) phase.

Since MSA has been shown to induce apoptotic cell death in a number of prostate cancer cell lines [15,16], we set out to examine the effect of MSA on apoptosis in PC-3M cells. PC-3M is a metastatic subline derived from PC-3. We chose PC-3M because it has the highest level of survivin expression among the cell lines we have tested (data not shown). Cells were treated with 5 μM MSA for 48 and 66 hr and the extent of apoptosis was quantified by flow cytometric analysis of cells labeled with propidium iodide (PI). This dose of selenium has been shown to be achievable in human subjects taking selenium supplementation [17]. As shown in Fig 2, only 0.9% of the control cells were positively labeled with PI. In cells treated with MSA, the proportion of apoptotic cells increased to 3.6% by 48 hr, and to 14.7% by 72 hr. These results showed that MSA was capable of inducing apoptosis in PC-3M cells, but the effect was modest and a long treatment (>48 hr) was needed to achieve a significant induction of apoptosis.

**Figure 3:**
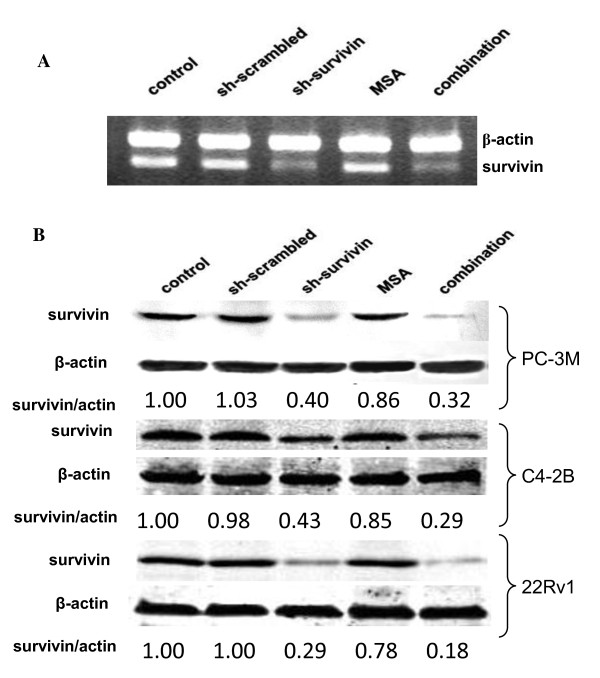
**Survivin expression in various prostate cancer cells treated with MSA, sh-survivin, or the combination**. A, RT-PCR analysis in PC-3M cells. B, Western blot analysis in PC-3M, C4-2B, and 22Rv1 cells. The band intensity was quantified by volume densitometry and normalized to that of β-actin. The results are expressed as fold induction over untreated.

As a member of the IAP family, the best known function of survivin is to protect the cells from undergoing apoptosis. It has been shown that selenium decreased survivin expression in several prostate cancer cell lines, including LNCaP, PC-3, and C4-2 [18]. We decided to examine survivin expression in PC-3M cells following MSA treatment. In contrast to what have been observed in other cell lines, both semi-quantitative RT-PCR and Western blotting analysis showed that treatment with 5 μM MSA for 66 hr did not influence survivin expression in PC-3M cells (Fig. 3A &3B), indicating the induction of apoptosis by MSA in these cells is not mediated by decreasing survivin expression. In addition to PC-3M cells, we analyzed survivin expression in C4-2B and 22Rv1 prostate cancer cells following MSA treatment and the results were similar (Fig. 3B).

### Survivin gene silencing sensitizes prostate cancer cells to growth inhibition by MSA

Based on the data from the previous experiments, we hypothesized that survivin knockdown will sensitize prostate cancer cells to growth inhibition by MSA. To test this hypothesis, we designed a plasmid-based shRNA vector for survivin gene silencing and tested its efficacy. As shown in Fig. 3A, sh-survivin effectively reduced the level of the survivin transcript, whereas the scrambled shRNA had no effect. Western blotting analysis confirmed the down-regulation of survivin expression by sh-survivin in all 3 cell lines (Fig. 3B). Quantitation by volume densitometry showed the knockdown efficiency at the protein level is in the range of 60-70% in these cell lines.

**Figure 4:**
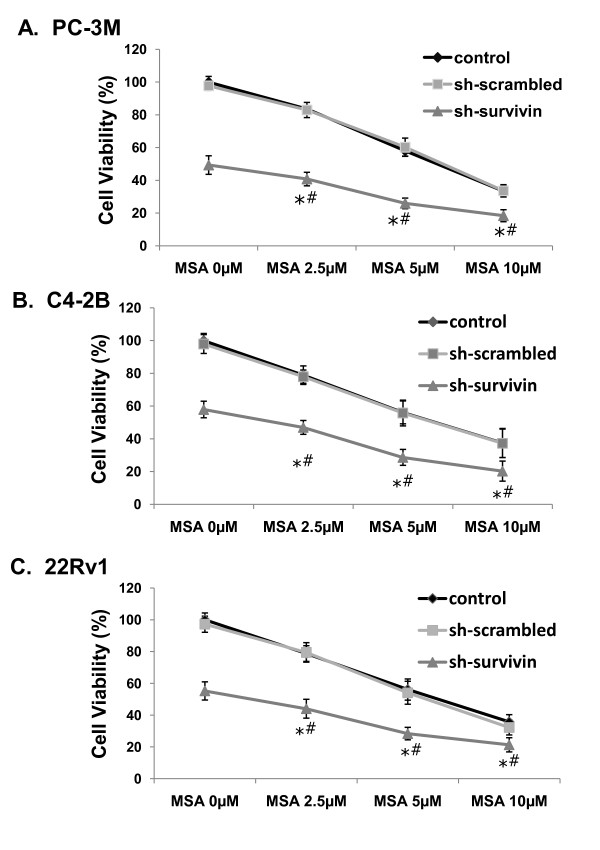
**Growth inhibition by survivin knockdown and MSA treatment**. PC-3M, C4-2B, and 22Rv1 cells were transfected with the shRNAs and treated with various concentrations of MSA for 66 hr. Cell numbers were quantitated by the MTT assay. The viability of the mock-transfected control cells were set arbitrarily to 100% and the viabilities of other samples were expressed as percentage of the control. The data presented are mean ± SD (n = 18). Control, mock-tranfected cells. Sh-scrambled, cells transfected with sh-scrambled. Sh-survivin, cells transfected with sh-survivin. *, *P *< 0.01 vs MSA alone; #, *P *< 0.01 vs sh-survivin alone.

We next quantitated changes in cell viability after cells were transfected with the shRNAs and treated with various concentrations of MSA for 66 hr. The results are shown in Fig. 4. Cells transfected with sh-scrambled have similar viability as the mock-transfected control cells under all circumstances. Silencing of survivin reduced cell viability by 50-60% (*P *< 0.01). In the absence of sh-survivin, MSA reduced the cell viability in a dose-dependent manner in all three cell lines. However, cells transfected with sh-survivin are significantly more sensitive to MSA growth inhibition, manifested by the marked downward shift of the dose-response curves. In addition, cells transfected with sh-survivin and treated with MSA, the decrease in cell viability was significantly greater than sh-survivin alone (*P *< 0.01).

### Survivin gene silencing enhances selenium's efficacy on apoptosis induction

**Figure 5:**
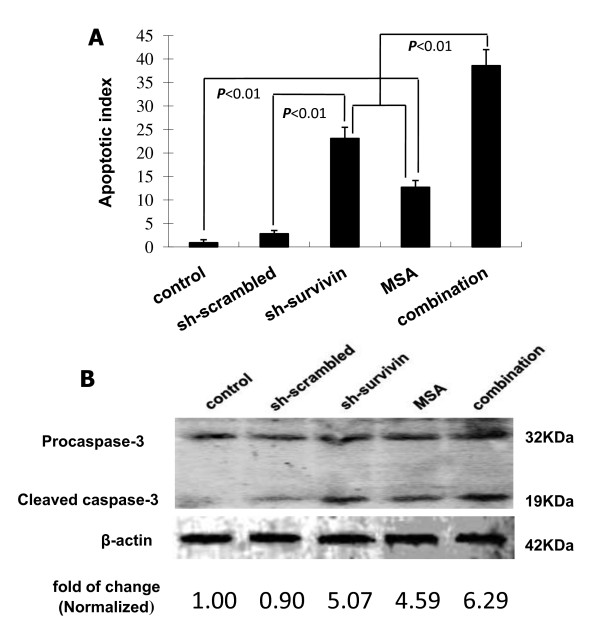
**Apoptosis induction by survivin knockdown and MSA treatment in PC-3M cells**. Cells were transfected with the shRNAs and treated with 5 μM MSA for 66 hr. A, Quantitation of apoptotic cells by PI staining and flow cytometry analysis. Apoptosis index was calculated as percentage of cells in the sub-G1 population on the DNA histogram. The data presented are mean ± SD (n = 3). The values are 0.9% ± 0.65%, 2.8% ± 0.71%, 23.1 ± 2.36%, 12.7 ± 1.45%, and 38.6 ± 2.87%, respectively. B, Western blot analyses of caspase-3. β-actin was used as the loading control. B. Quantitation of cleaved caspase-3 by volume densitometry. The intensity of the cleaved caspase-3 band was quantified by volume densitometry and normalized to that of β-actin. The result was expressed as fold to control.

Next, we analyzed the effect of apoptosis induction by sh-survivin and MSA in PC-3M cells by flow cytometry. Transfection and MSA treatment were carried out as described in the previous section. Very few PI-positive cells were found in untreated cells or in cells transfected with sh-scrambled (Fig. 5A). Silencing of survivin resulted in a robust increase of apoptotic cells, confirming the antiapoptotic role of survivin. The combination of survivin knockdown and MSA induced apoptosis more effectively than either alone (*P *< 0.01). In addition, AO/EB staining was performed to visualize the apoptotic cells and similar results were obtained (see additional file-2 for detail).

Caspases are important mediators of apoptosis. Activation of caspase-3 by enzymatic cleavage is a hallmark event in caspase-dependent apoptosis. We investigated caspase-3 activation by Western blotting and the results are shown in Fig. 5B. Cleaved caspase-3 (19 KDa) was induced similarly by sh-survivin and MSA, whereas their combination led to a modestly but significantly stronger activation (*P *< 0.05). The changes in caspase-3 activation did not involve changes in gene expression, as the band intensity for procaspase-3 (32 KDa) was not changed by either sh-survivin, or MSA, or their combination. The modest increase in caspase-3 activation in the combined group suggest that the synergistic effect on apoptosis induction could also be mediated by caspase-independent mechanisms, since survivin has also been shown to inhibit both caspase-independent apoptosis [19]. In general, the apoptosis data are in agreement with the MTT results, showing that survivin gene knockdown greatly enhances the anticancer effect of selenium in prostate cancer cells.

### Inhibition of tumor growth *in vivo *by survivin knockdown and selenium

**Figure 6:**
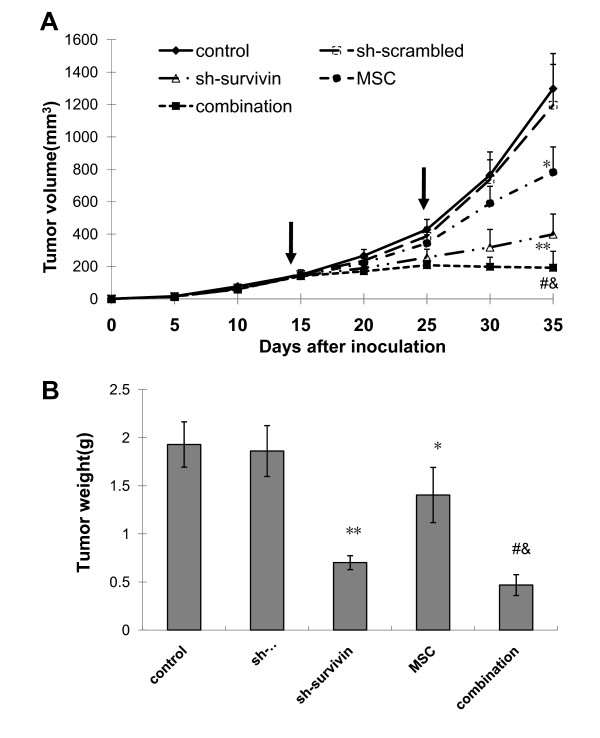
**Inhibition the growth of PC-3M xenografts by survivin knockdown and MSA**. The shRNA plasmids were transfected by electroporation twice at day 15 and 25, as indicated by the arrows. A, tumor volume. B, final tumor weight. The data presented are mean ± SD (n = 6).*, P < 0.05 vs control; **, P < 0.05 vs sh-scrambled; #, P < 0.05 vs sh-survivin; &, P < 0.05 vs MSC group.

A xenograft nude mouse tumor model with subcutaneously implanted PC-3M cells was used to investigate the effects of survivin knockdown and selenium *in vivo*. Fifteen days after injection, the tumors reached an average volume of 122.5 ± 15.36 mm^3^. The animals were then randomly divided into 5 groups receiving PBS, sh-scrambled, sh-survivin, MSC, or the combination of MSC and sh-survivin, respectively. MSC was administrated at a dose of 100 μg/mouse/day through an oral route and the shRNA plasmids were delivered by electroporation twice during the experiment, first at day 15 and then at day 25. As shown in Fig. 6A, tumor xenografts in the control and the scrambled groups grew at similar rates, whereas xenografts in all 3 treatment groups demonstrated reduced growth by day 30, when compared to their respective controls (*P *< 0.05). Consistent with the *in vitro *results, selenium by itself has a modest inhibitory effect on the growth of PC-3M xenografts, but the combination with survivin knockdown achieved a robust growth inhibition. In fact, tumors in the combination group maintained their original sizes after treatment, and were significantly smaller than those in the single treatment groups by day 35 (*P *< 0.05). Similar conclusions can be reached by examining the final tumor weight (Fig. 6B). These results demonstrate a strong inhibitory effect on tumor growth *in vivo *by the combination of survivin knockdown and selenium treatment.

**Figure 7:**
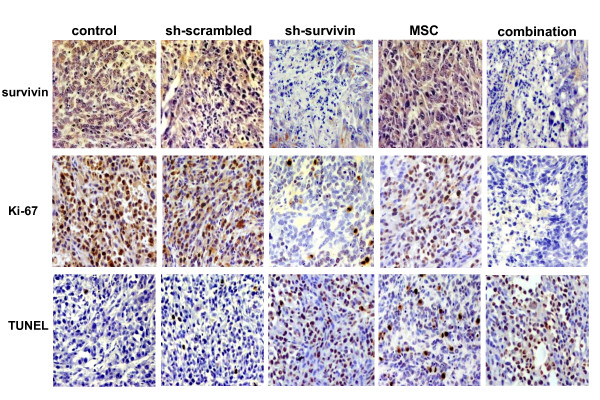
**Immunohistochemical staining in PC-3M tumor xenografts**. (A) survivin expression, (B) Ki-67 expression, and (C) TUNEL assay. The images were taken at 200 × magnification.

To explore the mechanisms of tumor inhibition *in vivo*, tumors were collected after the animals were sacrificed. The expression of survivin was first analyzed by IHC staining. As shown in Fig. 7A, the staining results showed that the electroporation-based delivery method was effective as survivin was down-regulated by sh-survivin. MSC has no effect on the expression or the cellular localization of survivin. Cell proliferation was quantitated by IHC staining of Ki-67 (Fig. 7B) and apoptotic cells were identified by the TUNEL assay (Fig. 7C). Tumors from the control or the sh-scrambled groups have proliferating cells (Ki-67 positive) and less apoptotic cells (TUNEL positive) than those in the sh-survivin or MSC. The lowest number of proliferating cells and highest number of apoptotic cells were found in tumors from the combination group. Quantitative analyses of the stained slides were performed and the results are summarized in Table 2. In tumors receiving sh-survivin or MSC, the proliferation index was significantly lower (*P *< 0.01), and the apoptotic index was significantly higher (*P *< 0.01), when compared with the control tumors. Once again, the combination group was more effective in blocking tumor growth and inducing cell death than either of the single treatment groups (*P *< 0.01). These results confirmed our *in vitro *observations and showed survivin knockdown greatly enhanced the efficacy of selenium in inhibiting the growth of prostate cancer cells *in vivo*.

**Table 2:** Cell proliferation and apoptosis indices from PC-3M xenografts.

Group (n = 6)	Proliferation index	Apoptotic index
control	86.75 ± 9.43	3.64 ± 1.66
sh-scrambled	81.64 ± 9.25	4.45 ± 1.86
sh-survivin	16.82 ± 5.32*	31.3 ± 3.64*
MSC	56.2 ± 8.65*	14.7 ± 2.23*
Combination	6.8 ± 1.64*^#^	48.6 ± 2.67*^#^

*, *P *< 0.01 vs sh-scrambled; #, *P *< 0.01 vs sh-survivin or MSC group.

## Discussion

Soon after the development of the RNA interference (RNAi) technology a few years ago, the potential of using this highly specific and powerful gene silencing approach in treating cancer has generated a great deal of interest. Obviously, the key to the success of this approach is to identify a gene which is expressed universally in cancer cells, but not in normal cells. Survivin appears to be such a candidate. As a member of the IAP family, survivin has been reported to be up-regulated in human cancers of various origins. Consistent with previous reports [5,20], we found in the present study that survivin is highly expressed in the majority of cancerous prostate tissues, but not in normal prostatic tissues. Silencing of survivin expression in PC-3M cells by a specific shRNA led to decreased cell growth and increased cell death. Elevated survivin expression has been associated with resistance to chemotherapeutic agents [21-24]. Therefore, targeted suppression of survivin expression represents a potential therapeutic strategy for prostate cancer, either being used alone or in a neoadjuvant setting.

Several potential reasons have been discussed to explain the discrepancy of the findings in SELECT and the NPC trial. One important consideration is the baseline selenium level. The NPC trial showed that the protective effect of selenium was limited to patients with baseline serum selenium in the lower 2 tertiles [25]. The average baseline selenium level of the participants in SELECT was much higher than that observed in the NPC study. In fact, 78% of men in SELECT had baseline selenium above the range that selenium provided protection in the NPC trial (<121.6 ng/ml) [11]. Another important consideration is how selenium exerts its anticancer activity. By using a nested case-control design within a prospective study (the Physicians' Health Study) among 586 men diagnosed with prostate cancer during 13 years of follow-up and 577 control subjects, Li et al. showed a ~50% reduction in risk of advanced prostate cancer in individuals within the highest quintile of selenium level compared to those within the lowest quintile [26]. Such association was not observed for localized prostate cancer, suggesting that selenium might function by slowing down tumor progression [26]. In view of the above information, we believe that the negative finding by SELECT should not be simply interpreted as selenium is ineffective against prostate cancer; instead, it raises the hypothesis of whether selenium might only be effective only in selected subsets of men with lower selenium levels at baseline.

The formulation and dose of selenium used in the SELECT study have also been hot topics of debate. The compounds used in the current study, MSA and MSC, are monomethylated forms of selenium and are considered second-generation selenium compounds. Metabolically, they are very different from selenomethionine, the formulation used in the SELECT. MSA and MSC can be easily converted to methylselenol, which is considered to be the critical metabolite for the anticancer activity of selenium [14,27]. Selenomethionine, on the other hand, can be incorporated nonspecifically into proteins in place of methionine [14]. Due to its compartmentation into tissue proteins, selenomethionine is not as readily available as MSA or MSC for further metabolism. The metabolism of selenomethionine to methylselenol requires at least 5 transsulfuration steps and the action of thiol methyltransferase [12-14]. Studies published prior to and after the start of the SELECT study have showed that MSA and MSC have stronger anticancer activities than selenomethionine [28-30].

Several studies have shown that selenium induces growth arrest and cell death in prostate cancer cells and inhibits the growth of prostate cancer xenografts [15,16,31,32], leading support to the idea that selenium could be used in the treatment of prostate cancer. In the present study, we confirmed the anticancer activity of selenium in the androgen-independent human prostate cancer cells. In contrast to a report that selenium suppresses survivin expression in a number of PCa cell lines, including the parental PC-3 and LNCaP lines [18], our *in vitro *and *in vivo *data both showed that MSA treatment had little or no effect on survivin expression in PC-3M, C4-2B, and 22Rv1 cells. This is consistent with results published by Azrak et al showing at similar doses, selenium treatment did not affect survivin expression in mouse prostate cancer cells or ovarian cancer cells [33,34]. When used alone, MSA had a modest effect on growth inhibition and apoptosis induction in PC-3M cells cultured *in vitro*. Similarly, MSC inhibited the growth of PC-3M xenografts but the efficacy appears to be inferior to its inhibitory effect against PC-3 xenografts [29]. The insensitivity of PC-3M cells to selenium could be related to its inability to suppress survivin expression in these cells. In support of the notion, we found that the effect of selenium was greatly enhanced when survivin expression was silenced. Indeed, in nude mice bearing PC-3M xenografts, survivin knockdown in combination with selenium treatment stopped tumor growth completely. In the study by Azrak et al which showed MSA did not inhibit survivin expression in skov3 ovarian cancer cells, survivin knockdown greatly enhanced the efficacy of combination therapy by MSA and paclitaxel, converting the interaction between these two agents from antagonistic to synergistic [34].

## Conclusions

Survivin was highly expressed in clinical prostate cancers but not in normal prostates. In prostate cancer cells where selenium had no effect on survivin expression, growth inhibition and apoptosis induction effects of selenium were modest. Both the *in vitro *and *in vivo *efficacies of selenium were greatly enhanced when survivin expression was silenced. The current study suffers from the limitation that the results are obtained mainly from cell culture and animal experiments. Nonetheless, the results suggest a potential mechanism of cancer cell resistance to the cytotoxic effect of selenium compounds and a strategy to improve the efficacy of selenium. This is important, especially in the context of the negative findings of the SELECT trial with regard to the anticancer activity of selenium. Further preclinical studies are needed to confirm the findings before these results can be incorporated into clinical practice.

## Competing interests

The authors declare that they have no competing interests.

## Authors' contributions

This study was designed by XL, YD, XZ, and HZ. XL carried out the majority of the experiments and performed the data analysis. RG helped with the animal experiments, data analysis, and manuscript preparation. YD provided assistance with data analysis, result interpretation, and manuscript preparation. LG helped with shRNA design and YZ with flow cytometry analysis. LZ and XZ were responsible for patient sample collection and coordination of the study. The manuscript was written by XL and HZ. HZ also contributed to data analysis and interpretation. All authors have read and approved the final manuscript.

## Pre-publication history

The pre-publication history for this paper can be accessed here:

http://www.biomedcentral.com/1471-2407/10/418/prepub

## Supplementary Material

Additional file 1 **This file contains the original data used for the Pearson correlation analysis for survivin expression and Gleason score**. It also contains the PSA measurements. Click here for file

Additional file 2 **This file contains additional figure for apoptosis detection using AO/EB staining as described in the text**. Click here for file

## References

[B1] Jemal A Siegel R Ward E Murray T Xu J Smigal C Thun MJ Cancer statistics, 2006 CA Cancer J Clin 2006 56 106 130 10.3322/canjclin.56.2.106 16514137

[B2] Klein EA Kupelian PA Localized prostate cancer: radiation or surgery? Urol Clin North Am 2003 30 315 30ix 10.1016/S0094-0143(02)00179-9 12735507

[B3] Altieri DC Marchisio PC Survivin apoptosis: an interloper between cell death and cell proliferation in cancer Lab Invest 1999 79 1327 1333 10576203

[B4] Altieri DC Survivin, versatile modulation of cell division and apoptosis in cancer Oncogene 2003 22 8581 8589 10.1038/sj.onc.1207113 14634620

[B5] Shariat SF Lotan Y Saboorian H Khoddami SM Roehrborn CG Slawin KM Ashfaq R Survivin expression is associated with features of biologically aggressive prostate carcinoma Cancer 2004 100 751 757 10.1002/cncr.20039 14770431

[B6] Brooks JD Metter EJ Chan DW Sokoll LJ Landis P Nelson WG Muller D Andres R Carter HB Plasma selenium level before diagnosis and the risk of prostate cancer development J Urol 2001 166 2034 2038 10.1016/S0022-5347(05)65500-0 11696701

[B7] Helzlsouer KJ Huang HY Alberg AJ Hoffman S Burke A Norkus EP Morris JS Comstock GW Association between alpha-tocopherol, gamma-tocopherol, selenium, and subsequent prostate cancer J Natl Cancer Inst 2000 92 2018 2023 10.1093/jnci/92.24.2018 11121464

[B8] Nomura AM Lee J Stemmermann GN Combs GF Jr Serum selenium and subsequent risk of prostate cancer Cancer Epidemiol Biomarkers Prev 2000 9 883 887 11008904

[B9] Clark LC Combs GF Jr Turnbull BW Slate EH Chalker DK Chow J Davis LS Glover RA Graham GF Gross EG Effects of selenium supplementation for cancer prevention in patients with carcinoma of the skin. A randomized controlled trial. Nutritional Prevention of Cancer Study Group JAMA 1996 276 1957 1963 10.1001/jama.276.24.1957 8971064

[B10] Duffield-Lillico AJ Dalkin BL Reid ME Turnbull BW Slate EH Jacobs ET Marshall JR Clark LC Selenium supplementation, baseline plasma selenium status and incidence of prostate cancer: an analysis of the complete treatment period of the Nutritional Prevention of Cancer Trial BJU Int 2003 91 608 612 10.1046/j.1464-410X.2003.04167.x 12699469

[B11] Lippman SM Klein EA Goodman PJ Lucia MS Thompson IM Ford LG Parnes HL Minasian LM Gaziano JM Hartline JA Effect of selenium and vitamin E on risk of prostate cancer and other cancers: the Selenium and Vitamin E Cancer Prevention Trial (SELECT) JAMA 2009 301 39 51 10.1001/jama.2008.864 19066370 PMC3682779

[B12] Ip C Ganther HE Activity of methylated forms of selenium in cancer prevention Cancer Res 1990 50 1206 1211 2105164

[B13] Ip C Hayes C Budnick RM Ganther HE Chemical form of selenium, critical metabolites, and cancer prevention Cancer Res 1991 51 595 600 1824684

[B14] Ip C Lessons from basic research in selenium and cancer prevention J Nutr 1998 128 1845 1854 9808633 10.1093/jn/128.11.1845

[B15] Yamaguchi K Uzzo RG Pimkina J Makhov P Golovine K Crispen P Kolenko VM Methylseleninic acid sensitizes prostate cancer cells to TRAIL-mediated apoptosis Oncogene 2005 24 5868 5877 10.1038/sj.onc.1208742 15897871

[B16] Zu K Ip C Synergy between selenium and vitamin E in apoptosis induction is associated with activation of distinctive initiator caspases in human prostate cancer cells Cancer Res 2003 63 6988 6995 14583501

[B17] Burk RF Hill KE Motley AK Austin LM Norsworthy BK Deletion of selenoprotein P upregulates urinary selenium excretion and depresses whole-body selenium content Biochim Biophys Acta 2006 1760 1789 1793 17014962 10.1016/j.bbagen.2006.08.010 PMC1761947

[B18] Chun JY Hu Y Pinder E Wu J Li F Gao AC Selenium inhibition of survivin expression by preventing Sp1 binding to its promoter Mol Cancer Ther 2007 6 2572 2580 10.1158/1535-7163.MCT-07-0172 17876054 PMC2821810

[B19] Liu T Brouha B Grossman D Rapid induction of mitochondrial events and caspase-independent apoptosis in Survivin-targeted melanoma cells Oncogene 2004 23 39 48 10.1038/sj.onc.1206978 14712209 PMC2292403

[B20] Kaur P Kallakury BS Sheehan CE Fisher HA Kaufman RP Jr Ross JS Survivin and Bcl-2 expression in prostatic adenocarcinomas Arch Pathol Lab Med 2004 128 39 43 14692814 10.5858/2004-128-39-SABEIP

[B21] Virrey JJ Guan S Li W Schonthal AH Chen TC Hofman FM Increased survivin expression confers chemoresistance to tumor-associated endothelial cells Am J Pathol 2008 173 575 585 10.2353/ajpath.2008.071079 18599610 PMC2475793

[B22] Zhang M Mukherjee N Bermudez RS Latham DE Delaney MA Zietman AL Shipley WU Chakravarti A Adenovirus-mediated inhibition of survivin expression sensitizes human prostate cancer cells to paclitaxel in vitro and in vivo Prostate 2005 64 293 302 10.1002/pros.20263 15754318

[B23] Zhang M Latham DE Delaney MA Chakravarti A Survivin mediates resistance to antiandrogen therapy in prostate cancer Oncogene 2005 24 2474 2482 10.1038/sj.onc.1208490 15735703

[B24] Yang H Fu JH Hu Y Huang WZ Zheng B Wang G Zhang X Wen J Influence of SiRNA Targeting Survivin on Chemosensitivity of H460/cDDP Lung Cancer Cells J Int Med Res 2008 36 734 747 18652770 10.1177/147323000803600416

[B25] Duffield-Lillico AJ Reid ME Turnbull BW Combs GF Jr Slate EH Fischbach LA Marshall JR Clark LC Baseline characteristics and the effect of selenium supplementation on cancer incidence in a randomized clinical trial: A summary report of the nuritional prevention of cancer trial Cancer Epidemiol Biomarkers Prev 2002 11 630 639 12101110

[B26] Li H Stampfer MJ Giovannucci EL Morris JS Willett WC Gaziano JM Ma J A prospective study of plasma selenium levels and prostate cancer risk J Natl Cancer Inst 2004 96 696 703 10.1093/jnci/djh125 15126606

[B27] Ip C Thompson HJ Zhu Z Ganther HE In vitro and in vivo studies of methylseleninic acid: evidence that a monomethylated selenium metabolite is critical for cancer chemoprevention Cancer Res 2000 60 2882 2886 10850432

[B28] Ip C Birringer M Block E Kotrebai M Tyson JF Uden PC Lisk DJ Chemical speciation influences comparative activity of selenium-enriched garlic and yeast in mammary cancer prevention J Agric Food Chem 2000 48 2062 2070 10.1021/jf000051f 10888499

[B29] Li GX Lee HJ Wang Z Hu H Liao JD Watts JC Combs GF Jr Lu J Superior in vivo inhibitory efficacy of methylseleninic acid against human prostate cancer over selenomethionine or selenite Carcinogenesis 2008 29 1005 1012 10.1093/carcin/bgn007 18310093 PMC3312608

[B30] Medina D Thompson H Ganther H Ip C Se-methylselenocysteine: a new compound for chemoprevention of breast cancer Nutr Cancer 2001 40 12 17 10.1207/S15327914NC401_5 11799917

[B31] Dong Y Lee SO Zhang H Marshall J Gao AC Ip C Prostate specific antigen expression is down-regulated by selenium through disruption of androgen receptor signaling Cancer Res 2004 64 19 22 10.1158/0008-5472.CAN-03-2789 14729601

[B32] Dong Y Zhang H Gao AC Marshall JR Ip C Androgen receptor signaling intensity is a key factor in determining the sensitivity of prostate cancer cells to selenium inhibition of growth and cancer-specific biomarkers Mol Cancer Ther 2005 4 1047 1055 10.1158/1535-7163.MCT-05-0124 16020662

[B33] Azrak RG Frank CL Ling X Slocum HK Li F Foster BA Rustum YM The mechanism of methylselenocysteine and docetaxel synergistic activity in prostate cancer cells Mol Cancer Ther 2006 5 2540 2548 10.1158/1535-7163.MCT-05-0546 17041098 PMC2826137

[B34] Azrak RG Frank CL Ghadersohi A Rustum YM Silencing survivin results in synergy between methylseleninic acid and paclitaxel against skov3 ovarian cancer cells Cancer Biol Ther 2008 7 1901 1908 18981709 10.4161/cbt.7.12.6939

