# Supplementary material for: Survivin gene silencing sensitizes prostate cancer cells to selenium growth inhibition
Source: BMC Cancer. 2010 Aug 10;10:418. doi: 10.1186/1471-2407-10-418 (PMC2928796; doi:10.1186/1471-2407-10-418)
Supplement: Additional file 1 — This file contains the original data used for the Pearson correlation analysis for survivin expression and Gleason score. It also contains the PSA measurements. [file 1471-2407-10-418-S1.PDF]

Case Number	Gleason scores	survivin expression*	Age	PSA(ug/ml)
1	4	0	84	20
2	5	0	68	3.7
3	5	1	61	84.4
4	6	0	75	2
5	6	0	82	18
6	6	1	81	60.2
7	6	2	57	14.8
8	7	1	85	72.8
9	7	2	70	56.6
10	7	2	82	150
11	7	2	72	5.08
12	7	2	77	32
13	7	3	75	76.7
14	7	3	61	52
15	7	3	71	46.5
16	8	2	64	78.2
17	8	3	74	146
18	8	3	82	84.3
19	8	2	67	140
20	8	2	83	81.7
21	8	3	82	36.7
22	8	3	80	90
23	9	3	49	3
24	9	2	76	78.4
25	9	3	69	85
26	9	3	85	150
27	9	2	76	32.01
28	10	3	77	39

* Survivin expression is graded as:

Negative	0
low	1
moderate	2
high	3
