# Supplementary figures and images for: Survivin gene silencing sensitizes prostate cancer cells to selenium growth inhibition
Source: BMC Cancer. 2010 Aug 10;10:418. doi: 10.1186/1471-2407-10-418 (PMC2928796; doi:10.1186/1471-2407-10-418)

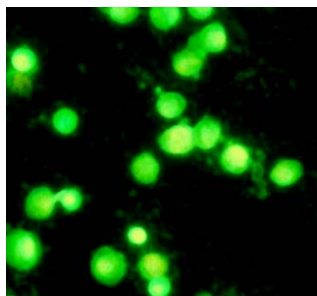

control

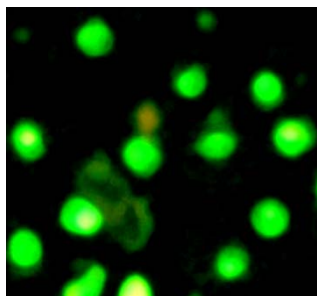

sh-scrambled

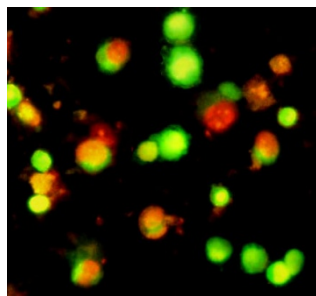

sh-survivin

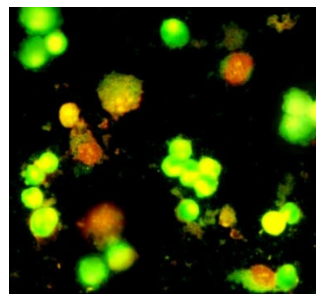

MSA

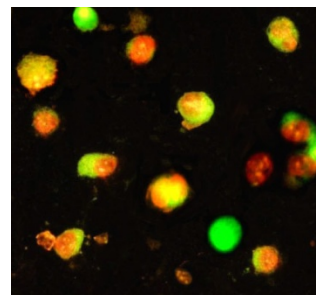

combination

Supplement: Additional file 2 — This file contains additional figure for apoptosis detection using AO/EB staining as described in the text. [file 1471-2407-10-418-S2.PDF]
